# Conventional Hemodynamic Resuscitation May Fail to Optimize Tissue Perfusion: An Observational Study on the Effects of Dobutamine, Enoximone, and Norepinephrine in Patients with Acute Myocardial Infarction Complicated by Cardiogenic Shock

**DOI:** 10.1371/journal.pone.0103978

**Published:** 2014-08-01

**Authors:** Corstiaan A. den Uil, Wim K. Lagrand, Martin van der Ent, Koen Nieman, Ard Struijs, Lucia S. D. Jewbali, Alina A. Constantinescu, Peter E. Spronk, Maarten L. Simoons

**Affiliations:** 1 Thoraxcenter, Erasmus Medical Center, Departments of Cardiology and Intensive Care Medicine, Rotterdam, the Netherlands; 2 Academic Medical Center, Department of Intensive Care Medicine, Amsterdam, the Netherlands; 3 Maasstad Hospital, Department of Cardiology, Rotterdam, the Netherlands; 4 Gelre Hospitals, Department of Intensive Care Medicine, Apeldoorn, the Netherlands; KRH Robert Koch Klinikum Gehrden, Germany

## Abstract

**Aim:**

To investigate the effects of inotropic agents on parameters of tissue perfusion in patients with cardiogenic shock.

**Methods and Results:**

Thirty patients with cardiogenic shock were included. Patients received dobutamine, enoximone, or norepinephrine. We performed hemodynamic measurements at baseline and after titration of the inotropic agent until cardiac index (CI) ≥2.5 L.min^−1^.m^−2^ or mixed-venous oxygen saturation (SvO_2_) ≥70% (dobutamine or enoximone), and mean arterial pressure (MAP) ≥70 mmHg (norepinephrine). As parameters of tissue perfusion, we measured central-peripheral temperature gradient (delta-T) and sublingual perfused capillary density (PCD). All patients reached predefined therapeutic targets. The inotropes did not significantly change delta-T. Dobutamine did not change PCD. Enoximone increased PCD (9.1 [8.9–10.2] vs. 11.4 [8.4–13.9] mm.mm^−2^; p<0.05), and norepinephrine tended to decrease PCD (9.8 [8.5–11.9] vs. 8.8 [8.2–9.6] mm.mm^−2^, p = 0.08). Fifteen patients (50%) died within 30 days after admission. Patients who had low final PCD (≤10.3 mm.mm^−2^; 64%) were more likely to die than patients who had preserved PCD (>10.3 mm.mm^−2^; mortality 72% vs. 17%, p = 0.003).

**Conclusion:**

This study demonstrates the effects of commonly used inotropic agents on parameters of tissue perfusion in patients with cardiogenic shock. Despite hemodynamic optimization, tissue perfusion was not sufficiently restored in most patients. In these patients, mortality was high. Interventions directed at improving microcirculation may eventually help bridging the gap between improved hemodynamics and dismal patient outcome in cardiogenic shock.

## Introduction

Patients with cardiogenic shock are at risk for impaired perfusion of organs and tissues. Low peripheral skin temperature as well as impaired sublingual tissue perfusion have been associated with multiple organ failure and adverse outcome in hemodynamically compromised patients [1], [2], [3], [4], [5]. Inotropic agents have the potential to maintain or restore adequate end-organ perfusion and function, but their use in heart failure has been associated with increased myocardial oxygen demand and cardiac arrhythmias [6]. Nevertheless, inotropic therapy is often considered necessary in patients with cardiogenic shock to improve the hemodynamic status; more specifically to increase cardiac output, to decrease pulmonary capillary wedge pressure, and to increase mean arterial pressure [7], [8], [9]. Pharmacologic treatment of patients with cardiogenic shock has several clinical challenges. First, the utility of inotropic therapy in restoring end-organ perfusion in patients with cardiogenic shock is based primarily on clinical experience rather than clinical trial data [6]. However, clinical examination may be inaccurate in identifying patients with low output states and impaired organ perfusion [10]. Second, global hemodynamic parameters do not reflect differential patterns of regional organ blood flow or compromised tissue perfusion of the splanchnic bed associated with shock states. For example, mean arterial blood pressure is often clinically used as a surrogate marker for tissue perfusion. However, not all patients with low output states present with hypotension and not all patients with hypotension have impaired organ perfusion [10], [11]. This is further demonstrated by the results of the ESCAPE trial that did not support the routine use of pulmonary artery catheter guided therapy in acute heart failure [12]. Therefore, there is a great need for techniques that directly assess tissue perfusion in patients at the bedside [13]. These novel techniques should provide guidance for the evaluation of existing and future vasoactive therapies for acute heart failure syndromes and may be the key to understand the interventions required to reduce the morbidity and mortality associated with multiple organ failure in patients with cardiogenic shock. We hypothesized that current hemodynamic resuscitation based on classic hemodynamic parameters would fail to improve microcirculation in at least a subset of patients. Aim of this pilot study was further to elucidate whether the effects of inotropic therapy on microcirculation could be monitored by parameters of tissue perfusion, including sublingual microcirculation as a surrogate for splanchnic perfusion.

## Methods

### Study design

This observational study was conducted at the Intensive Cardiac Care Unit of the Thoraxcenter, Erasmus University Medical Center, the Netherlands. The study population consisted of a subgroup of a cohort described previously [5]. All patients had acute myocardial infarction, complicated by cardiogenic shock. A patient was included in the current study when routine hemodynamic resuscitation maneuvers were performed and an investigator was present to perform the measurements. Cardiogenic shock was defined as echocardiographic evidence of cardiac dysfunction (poor left and/or right ventricular function or severe mitral regurgitation) and clinical signs of hypoperfusion (cold extremities, oliguria or altered mental state), after adequate fluid resuscitation, i.e. pulmonary capillary wedge pressure (PCWP) ≥18 mm Hg or no further increase of cardiac output after a fluid challenge. Patients with oral bleeding were excluded from the study since this would have hampered the measurements. The Erasmus MC medical ethical committee approved the protocol, and written informed consent was obtained from each patient or, in case of patients who were sedated, from a relative authorized to consent on behalf of such a patient.

### Hemodynamic monitoring

All patients were monitored with a radial artery catheter (arterial cannula with FloSwitch, Ohmeda, Swindon, UK) and a pulmonary artery catheter (Criticath SP5107H, Becton Dickinson, Sandy, UT, USA or CCOmbo, Edwards Lifesciences, Saint-Prex, Switzerland). Data collection included central body temperature (measured at the tip of the pulmonary artery catheter), heart rate (HR), mean arterial pressure (MAP), central venous pressure (CVP), PCWP, mean pulmonary artery pressure (PAP), cardiac index (CI), systemic vascular resistance (SVR), and mixed-venous oxygen saturation (SvO_2_). SVR was calculated as (MAP-CVP)*80/cardiac output. When no pulmonary artery catheter was present, we estimated cardiac index from central venous oxygen saturation (right atrium, Cuschieri formula [14]).

### Microcirculatory assessment and analysis

Central-peripheral temperature gradient (delta-T) was defined and calculated as the difference between central blood and skin temperature. Central blood temperature was measured with a pulmonary artery catheter or urinary bladder catheter. Skin temperature was measured with a probe sticked on the dorsum of the uncovered foot under constant room temperature (i.e. 21.0 degrees Celsius; temperature probe 170075; Ellab Inc., Centennial, CO, USA).

The Sidestream Dark Field (SDF) imaging device (MicroScan; Microvision Medical, Amsterdam, The Netherlands) was used to obtain 2-dimensional video images of sublingual microcirculatory blood flow. This technique has been described previously [15]. In short, the camera emits green light that is absorbed by red blood cells within microvessels. In this way, red blood cells are used as the contrast agent to visualize sublingual blood flow in patent microvessels. Per time point, three steady high-quality video sequences of at least 20 secs duration were obtained and stored on a laptop. Video files were then renamed using a randomly allocated number and analyzed blindly. Quantification of the images was done using dedicated software (Automated Vascular Analysis (AVA) 3.0, MicrovisionMedical, Amsterdam, the Netherlands). Perfused capillary density (PCD) was calculated by automated measuring the total length of perfused capillaries divided by image area. Capillaries were defined as microvessels with a diameter less than 20 µm. Capillaries were regarded as perfused if they had either of the following flow classifications obtained by visual inspection: sluggish, continuous or hyperdynamic [16]. Since SDF imaging enables visualization of flowing intravascular erythrocytes rather than microvessel walls, an increase in PCD was regarded as capillary recruitment. SDF imaging has been shown to be superior to conventional orthogonal polarization spectral imaging [15]. The AVA software has been validated using videos of simulated microvascular flow as well as using videos obtained from intensive care patients [17], [18]. Hubble et al reported an intraobserver variability of <6% and an interobserver variability of <13% for all measures of small vessels (capillaries) [19]. PCD measurements are reproducible with low variability in patients with severe heart failure [20].

### Study protocol

All measurements were performed within 24 hours after hospital admission. To minimize the effect of regression to the mean due to spontaneous variation in microcirculatory perfusion, two series of baseline measurements were performed with a time interval of 15 minutes. Values from both baseline measurements were averaged to obtain single baseline values. Reference values for sublingual PCD in control patients (i.e. patients awaiting cardiac surgery who were not in shock) have been reported previously, i.e. ≥11.7 mm.mm^−2^ (≥2.5 percentile) [21]. Reference values for sublingual PCD, associated with poor outcome in patients with cardiogenic shock, were reported as ≤10.3 mm.mm^−2^ [5].

The decision to start dobutamine, enoximone or norepinephrine was made by the attending physician. Dobutamine or enoximone, depending on preference of the attending physician, were given when cardiac index (CI) was <2.2 L.min^−1^.m^−2^ or when mixed-venous oxygen saturation (SvO_2_) was <65%. Both inotropic agents were only administered when mean arterial pressure (MAP) was ≥60 mm Hg. Dobutamine and enoximone were up titrated until the following targets had been reached: CI ≥2.5 L.min^−1^.m^−2^ or SvO_2_ ≥70%. Norepinephrine was given to patients when (MAP) was <60 mm Hg, independent of CI or SvO_2_. Norepinephrine was up titrated until MAP ≥70 mmHg. After the second baseline measurement, the inotropic agent was given as a bolus equal to the volume of the used intravenous line. Immediately thereafter, a continuous intravenous infusion was started and titrated. All measurements were repeated 10 minutes (dobutamine or norepinephrine) or 30 minutes (enoximone) after the maximum infusion rate had been started. During the study period, dosages of other vasoactive medications were kept constant. All components of the Sequential Organ Failure Assessment (SOFA) score were calculated, with the exception of the central nervous system parameters, because the majority of the patients received central nervous system depressant drugs at the time of evaluation.

### Statistical analysis

Categorical variables are presented as absolute numbers with percentages. All continuous variables are presented as median and interquartile range (IQR). Differences between groups were tested with the chi-square test, the Mann-Whitney test or the Kruskal-Wallis test, when appropriate. Changes between time points were tested with the Wilcoxon signed ranks test. Correlations between variables were investigated with Spearman's correlation test. Thirty-day mortality was stratified according to low PCD (ie, ≤10.3 mm.mm^−2^) or preserved PCD (ie, >10.3 mm.mm^−2^), according to a previous publication [5]. A p-value<0.05 was regarded statistically significant.

## Results

### Study population

Thirty patients with cardiogenic shock were included in this study, in whom 33 measurements were performed (Table 1). Fourteen patients received dobutamine (dobu), 10 patients received enoximone (enox), and 9 patients received norepinephrine (nor). There were no differences in baseline characteristics between the groups (Table 1). The following maximum dosages of intropic agents were administered: 5.0 [5.0–5.6] µg.kg^−1^.min^−1^ (dobu), 2.0 [2.0–2.0] µg.kg^−1^.min^−1^ (enox), and 0.12 [0.04–0.21] µg.kg^−1^.min^−1^ (nor). Predefined hemodynamic targets for the administered vaso-active medications were reached after 60 [56–75] minutes (dobu), 85 [60–131] minutes (enox), and 75 [35–78] minutes (nor). No inotrope-induced arrhythmias occurred during execution of the study.

**Table 1:** Baseline characteristics of the study population.

Characteristic	Dobutamine (n = 14)	Enoximone (n = 10)	Norepinephrine (n = 9)	P-value
**Age**, years	64 [49–74]	64 [52–71]	62 [51–80]	NS
**Male**	8 (57%)	5 (50%)	5 (56%)	NS
**Medical history**				
AMI	4 (29%)	5 (50%)	2 (22%)	NS
PCI	3 (21%)	2 (20%)	1 (11%)	NS
CABG	2 (14%)	0 (0%)	2 (22%)	NS
Chronic heart failure	2 (14%)	4 (40%)	3 (33%)	NS
TIA/CVA	1 (7%)	2 (20%)	3 (33%)	NS
Peripheral arterial disease	2 (14%)	2 (20%)	3 (33%)	NS
Chronic kidney disease	2 (14%)	2 (20%)	2 (22%)	NS
COPD	2 (14%)	1 (10%)	1 (11%)	NS
**CV risk factors**				
Hypertension	4 (29%)	4 (40%)	5 (56%)	NS
Current smoking	2 (14%)	1 (10%)	4 (44%)	NS
Dyslipidaemia	5 (36%)	4 (40%)	2 (22%)	NS
Diabetes mellitus	6 (43%)	6 (60%)	3 (33%)	NS
**Laboratory**				
NT-proBNP, pmol.L^−1^	750 [186–1124]	527 [443–938]	1629 [393–4736]	NS
C-reactive protein, mg/L	52 [14–139]	29 [14–90]	91 [25–129]	NS
White blood cell count, *10E9/L	10.8 [9.5–12.8]	10.7 [9.6–15.3]	11.5 [8.2–19]	NS
**SOFA score (total)**	5 [3–6]	6 [5–7]	7 [5–8]	NS
Respiratory	1 [1–2]	2 [1–2]	1 [1–2]	NS
Coagulation	0 [0–0]	0 [0–0]	0 [0–1]	NS
Liver	0 [0–0]	0 [0–0]	0 [0–1]	NS
Cardiovascular	2 [2–3]	3 [2–3]	3 [2–4]	NS
Renal	0 [0–1]	1 [0–1]	1 [0–2]	NS
**Echocardiography**				
LVEF <30%	12 (86%)	9 (90%)	7 (78%)	NS
Severe MR	3 (21%)	4 (40%)	2 (22%)	NS
Poor RVF	2 (14%)	0 (0%)	0 (0%)	NS
**Mechanical ventilation**	10 (71%)	6 (60%)	8 (89%)	NS
**Concomitant nitroglycerin**	2 (14%)	1 (10%)	1 (11%)	NS
**Concomitant norepinephrine**	5 (36%)	5 (50%)	-	NS
**CVVH**	1 (7%)	0 (0%)	1 (11%)	NS
**Revascularization**				
PCI	11 (79%)	7 (70%)	5 (56%)	NS
CABG	0 (0%)	1 (10%)	0 (0%)	NS
**IABP counterpulsation**	7 (50%)	7 (70%)	4 (44%)	NS
**Timing of baseline measurements**				
Time from AMI diagnosis (h)	17 [5–21]	16 [6–23]	9 [7–18]	NS
Time from shock onset (h)	5 [4–7]	5 [3–6]	7 [5–9]	NS

Abbreviations: AMI, acute myocardial infarction; PCI, percutaneous coronary intervention; CABG, coronary artery bypass grafting; TIA, transient ischemic attack; CVA, cerebrovascular accident; COPD, chronic obstructive pulmonary disease; CV, cardiovascular; NT-proBNP, N-terminal-pro B-type natriuretic peptide; LVEF, left ventricular ejection fraction; MR, mitral regurgitation; RVF, right ventricular function; CVVH, continuous venovenous hemofiltration; IABP, intra-aortic balloon pump.

Values represent median [interquartile range] or number (percentage). P-values>0.05 (NS, non-significant) are not shown.

### Effects on systemic and pulmonary circulation

There were no significant differences in global hemodynamic or microcirculatory parameters between the two baseline measurements within each group, indicating that patients were in a stable condition before the inotropic agent was started. Baseline hemodynamic parameters were not significantly different between patients receiving dobutamine or enoximone. However, patients treated with norepinephrine had a lower baseline MAP (p = 0.006, Table 2).

**Table 2:** Parameters at baseline.

	Dobutamine (n = 14)	Enoximone (n = 10)	Norepinephrine (n = 9)	P-value
**HR, bpm**	84 [59–99]	91 [78–107]	101 [84–118]	NS
**MAP, mmHg**	66 [60–71]	68 [62–81]	55 [51–58]	0.006
**CVP, mmHg**	14 [11–17]	14 [9–16]	16 [12–19]	NS
**PCWP, mmHg**	19 [16–25]	23 [17–25]	20 [17–27]	NS
**MPAP, mmHg** a	27 [21–33]	33 [28–37]	28 [25–39]	NS
**CI, L.min^−1^.m^−2^**	2.2 [1.7–2.5]	1.9 [1.7–3.2]	2.6 [1.6–3.2]	NS
**SVR, dynes.sec.cm^−5^**	1182 [743–1502]	1057 [843–1246]	837 [555–1031]	NS
**SvO2, %**	64 [60–68]	62 [56–71]	67 [61–70]	NS
**Lactate, mmol.L^−1^**	1.4 [1.1–4.6]	1.3 [1.1–2.9]	2.1 [1.1–2.8]	NS
**Delta-T, °C**	6.6 [4.5–6.9]	6.1 [4.3–7.5]	6.2 [3.7–13.9]	NS
**PCD, mm.mm^−2^**	9.3 [8.0–10.1]	9.1 [8.9–10.2]	9.8 [8.6–11.9]	NS

Abbreviations: HR, heart rate; MAP, mean arterial pressure; CVP, central venous pressure; PCWP, pulmonary capillary wedge pressure; MPAP, mean pulmonary artery pressure; CI, cardiac index; SVR, systemic vascular resistance; SvO_2_, mixed-venous oxygen saturation; delta-T, central-peripheral temperature gradient; PCD, perfused capillary density.

Values represent median [interquartile range]. P-values≥0.05 (NS, non-significant) are not shown.

a A pulmonary artery catheter was present in 27/33 (82%) of the measurements.

**Figure 1:**
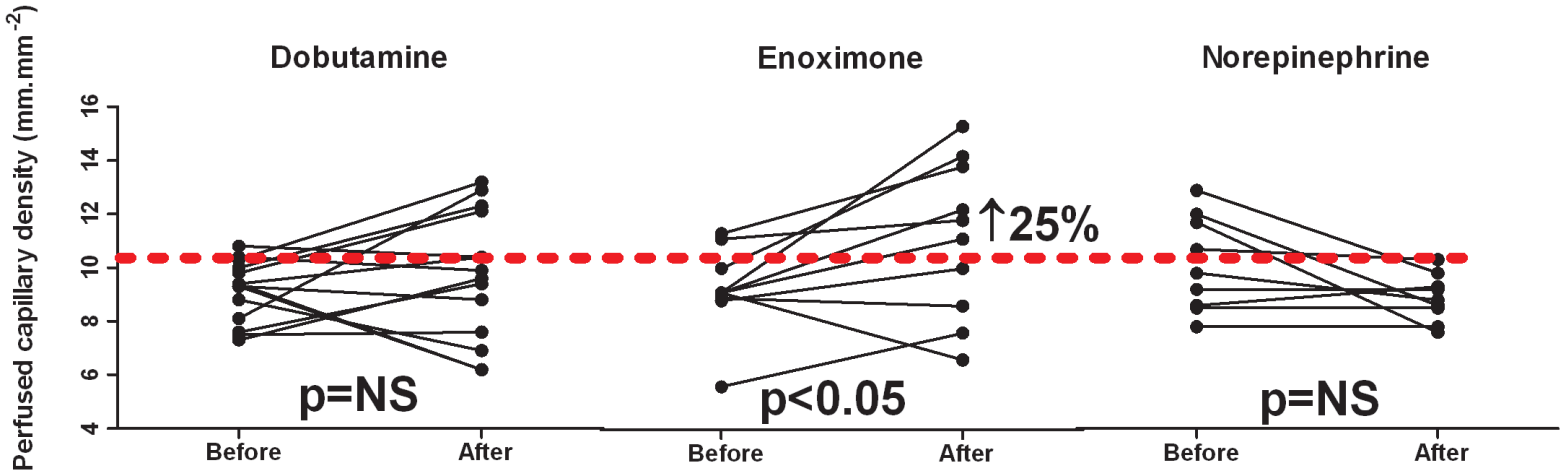
Changes in perfused capillary density following infusions of dobutamine (median 60 minutes), enoximone (median 85 minutes), and norepinephrine (median 75 minutes) at the individual patient level. ↑, increase of parameter following inotropic therapy in terms of percentage relative to baseline. The reference value of perfused capillary density is indicated by the red dashed line (ie, 10.3 mm.mm^−2^). Values greater than this reference are to be considered as preserved capillary density, values below or equal to this reference reflect impaired capillary density [5].

Absolute changes in parameters are shown in Table 3. Figure 1 shows the changes in PCD at the individual patient level.

**Table 3:** The effects of infusions of dobutamine (median 60 minutes), enoximone (median 85 minutes), and norepinephrine (median 75 minutes) on parameters of macro- and microcirculation.

	Dobutamine (n = 14)	Enoximone (n = 10)	Norepinephrine (n = 9)	P-value
**ΔHR, bpm**	+9 [0; +16]**	+4 [−11; +9]	+1 [−15; +4]	NS
**ΔMAP, mmHg**	+6 [−5; +21]	+8 [+1; +14]	+17 [+13; +32]**	NS
**ΔCVP, mmHg**	−1 [−3; +1]	−2 [−3; −1]*	+2 [−4; +4]	NS
**ΔPCWP, mmHg**	−2 [−4; −1]**	−2 [−3; −1]**	+5 [−1; +7]	NS
**ΔMPAP, mmHg** a	0 [−3; +3]	−1 [−9; 0]	+4 [−1; +7]	NS
**ΔCI, L.min^−1^.m^−2^**	+0.8 [+0.3; +1.4]**	+0.6 [−0.1; +1.5]	0.0 [−0.5; +0.1]	0.006
**ΔSVR, dynes.sec.cm^−5^**	−201 [−623; +220]	−119 [−491; +175]	+390 [+237; +505]*	0.03
**ΔSvO2, %**	+6 [+2; +12]**	0 [−3; +4]	0 [−3; +6]	0.04
**ΔLactate, mmol.L^−1^**	−0.4 [−2.5; −0.1]**	0.0 [−0.6; +0.2]	0.0 [−0.2; +0.5]	NS
**ΔDelta-T, °C**	−0.4 [−0.8; 0]	−1.1 [−1.9; +0.6]	0.0 [−2.2; +0.6]	NS
**ΔPCD, mm.mm^−2^**	+0.6 [−0.9; +2.3]	+2.0 [+0.5; +3.4]*	−0.4 [−3.3; 0.0]	0.01

Abbreviations: HR, heart rate; MAP, mean arterial pressure; CVP, central venous pressure; PCWP, pulmonary capillary wedge pressure; MPAP, mean pulmonary artery pressure; CI, cardiac index; SVR, systemic vascular resistance; SvO_2_, mixed-venous oxygen saturation; delta-T, central-peripheral temperature gradient; PCD, perfused capillary density.

Values represent median [interquartile range]. The p-value in the last column represents differences among groups. Asterisks indicate statistical significance versus baseline:

*, p<0.05;

**, p<0.01.

P-values>0.05 (NS, non-significant) are not shown.

a A pulmonary artery catheter was present in 27/33 (82%) of the measurements.

Dobutamine increased HR, decreased PCWP, increased CI and SvO2, and lowered lactate concentration. Enoximone decreased CVP en PCWP. Norepinephrine increased MAP and SVR. (Table 3).

### Effects on microcirculation

Baseline delta-T and PCD were not significantly different between patients receiving the various inotropic agents (Table 2). Figure 1 shows the changes in PCD at the individual patient level. Delta-T was not significantly changed by dobutamine, enoximone, or norepinephrine (Table 3). Dobutamine did not change PCD. Enoximone increased PCD (9.1 [8.9–10.2] vs. 11.4 [8.4–13.9] mm.mm^−2^; p<0.05), and norepinephrine tended to decrease PCD (9.8 [8.5–11.9] vs. 8.8 [8.2–9.6] mm.mm^−2^, p = 0.08, Table 3). After up titrating vasoactive therapy, 21 patients (64%) had still low PCD values, ie ≤10.3 mm.mm^−2^ (Figure 1).

### Correlations

Table 4 demonstrates modest correlations between (pooled) changes in global hemodynamic parameters and (pooled) changes in delta-T and PCD. Especially **i**n patients receiving norepinephrine, an increase in MAP was correlated to worsening of microcirculatory parameters (Spearman's rho = 0.60, p = 0.05 for delta-T and Spearman's rho = −0.70, p = 0.04 for PCD). Neither baseline delta-T nor baseline PCD were directly correlated to lactate levels at both time points. However, patients with low baseline PCD (< = 10.3 mm.mm^−2^) had higher baseline lactate levels (2.1 [1.3–3.8] vs. 1.1 [1.0–1.4] mmol/L, p = 0.03) as compared to patients with high baseline PCD. Changes in delta-T or PCD did not predict changes in lactate. Finally, changes in delta-T were correlated to changes in PCD (rho = −0.50, p = 0.01).

**Table 4:** Correlations between changes in pooled global hemodynamic and microcirculatory parameters.

	Δ*Delta-T, °C*	Δ*PCD, mm.mm^−2^*
**ΔMAP**	ρ = 0.52, p = 0.007	ρ = −0.44, p = 0.01
**ΔCI**	ρ = −0.48, p = 0.01	ρ = 0.38, p = 0.03
**ΔCVP**	ρ = 0.35, p = 0.08	ρ = −0.36, p = 0.05

ρ = Spearman's correlation coefficient.

### Outcome

**Figure 2:**
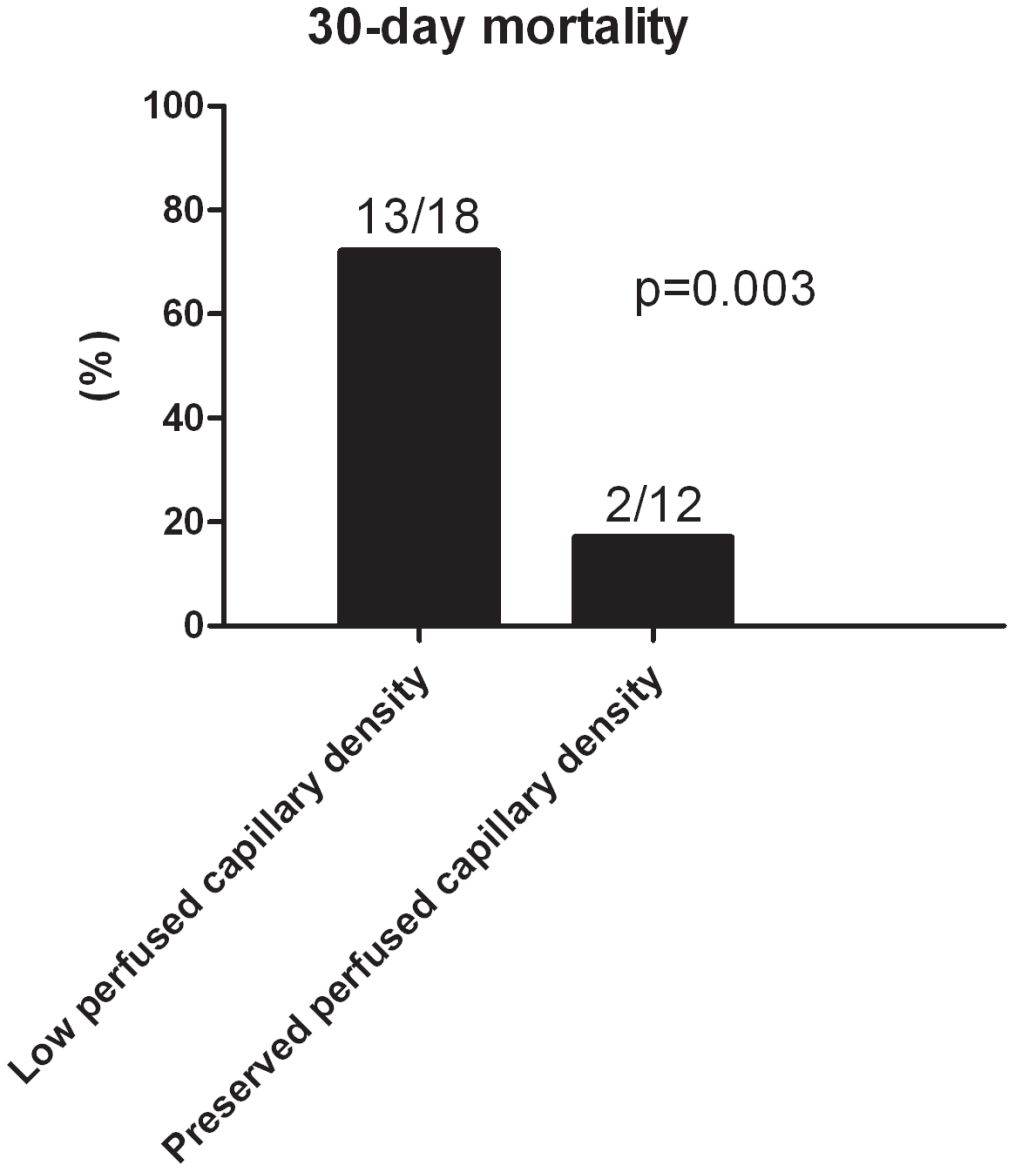
Thirty-day mortality according to final perfused capillary density. In the subgroup of patients (n = 18) with low perfused capillary density (ie, ≤10.3 mm.mm^−2^), 13 patients died, whereas in the subgroup of patients (n = 12) with preserved capillary density (ie, >10.3 mm.mm^−2^) 2 patients died (p = 0.003).

Fifteen patients (50%) died within 30 days after admission. Patients who had low final PCD (≤10.3 mm.mm^−2^; 64% of the patients) were more likely to die than patients who had preserved PCD (>10.3 mm.mm^−2^; mortality 72% vs. 17%, p = 0.003, Figure 2).

## Discussion

This study demonstrates the direct effects of various inotropes on the microcirculation measured at the bedside in patients with cardiogenic shock. There was a modest correlation between changes in global hemodynamic parameters and changes in delta-T and PCD. Despite optimization of global hemodynamics in all patients, 50% died. Patients with low final PCD were more likely to die than patients in whom tissue perfusion seemed to be restored.

### Effects on systemic and pulmonary circulation

Dobutamine and enoximone are the most commonly used intravenous inotropes for the management of cardiogenic shock in our center. Both agents are given to increase cardiac contractility by increasing intracellular levels of cyclic adenylate monophosphate (cAMP), although they affect cAMP by different mechanisms. Caldicott et al. compared the effects of enoximone with dobutamine in patients with severe heart failure following myocardial infarction [22]. These authors reported that both agents similarly increased cardiac output. Dobutamine, opposite to enoximone, also significantly increased heart rate and produced significantly more runs of supraventricular and ventricular tachycardia. Our findings agree with this report. Norepinephrine is an inotropic agent with high affinity for alfa-adrenergic receptors and acts therefore mainly as a vasopressor. Norepinephrine increases mean arterial pressure [23], and is therefore frequently used in the acute phase of cardiogenic shock to restore mean arterial pressure. In addition, norepinephrine may increase coronary blood flow in cardiogenic shock [24].

### Effects on microcirculation

Patients with severe heart failure and cardiogenic shock have impaired tissue perfusion and the severity of these abnormalities is clearly correlated with outcome [1], [5]. We recently reported the beneficial effects of intravenous nitrates in such patients [20], [25]. Lauten et al. performed serial measurements of sublingual microcirculation, at hospital admission and the day before discharge, in 27 patients with acute decompensated heart failure [26]. Using standard heart failure treatment, including diuretics, ACE-inhibitors, and betablockers, they reported a decrease in parameters of neurohumoral activation together with an increase in microvascular flow index, but correlations between changes in neurohormones and changes in microcirculation did not reach statistical significance. Salgado et al. found beneficial effects on sublingual microcirculation of angiotensin II inhibitors in patients with acute severe heart failure [27].

In our study of patients with cardiogenic shock, in whom inotropic support was judged necessary, there was no benefit of dobutamine on parameters of microcirculatory perfusion. De Backer et al. reported a modest improvement of sublingual capillary perfusion in patients with septic shock receiving dobutamine, but with large individual variation [28].

Enoximone improved capillary skin blood flow, measured by a laser Doppler technique, in a study in patients undergoing cardiopulmonary bypass surgery [29]. In addition, Kern et al. demonstrated a beneficial effect of enoximone on hepatosplanchnic oxygen consumption and on liver function in fluid-optimized septic shock patients [30]. Our study demonstrates a rather consistent increase in PCD, which favours the use of inodilators like enoximone over catecholamines.

The effects of norepinephrine on tissue perfusion are largely unknown. Most studies performed in patients with septic shock demonstrated no effect of norepinephrine on splanchnic perfusion [31], [32]. We are not aware of any reports on the direct effects of norepinephrine on the microcirculation in cardiogenic shock. Maier et al. recently investigated the response of the sublingual microcirculation to the pure alfa-adrenergic agonist phenylephrine during cardiopulmonary bypass surgery [33]. Increasing perfusion pressure from (mean) 47 to (mean) 68 mmHg using phenylephrine significantly decreased sublingual capillary microvascular flow index, whereas global tissue blood flow, measured with a laser Doppler flowmeter, increased. These findings were explained as phenylephrine-induced microcirculatory shunting. Indeed, using microspheres, Saxena and Verdouw demonstrated in the year 1985 the presence of arteriovenous anastomoses in the tongue [34]. In our study, norepinephrine tended to decrease sublingual PCD, where we should emphasize that in our patients receiving norepinephrine median baseline MAP was relatively high (55 mmHg).

### Correlations between macrocirculation and microcirculation

The apparent lack of strong correlation between cardiac index and microcirculatory parameters has been shown previously [28], [35]. This emphasizes that global hemodynamic parameters do not predict microcirculatory perfusion and underlines the importance of monitoring macro- as well as microcirculation in patients with cardiogenic shock [36]. In our study, increases in MAP or CVP correlated to deterioration of microcirculatory parameters, wherease an increase in CI weakly correlated to improvement of the microcirculation. These findings indicate that solely improving MAP (by means of norepinephrine) might not be a good strategy to improve tissue perfusion.

### Limitations

Several limitations of our study should be acknowledged. The small and heterogeneous sample size limits drawing strong conclusions. Next, the fact that our study was nonrandomized (especially dobutamine vs. enoximone) might have introduced bias. Finally, whether the effects of inotropic agents also apply to other microvascular beds, and whether efforts to directly improve the microcirculation will reduce cellular dysfunction, organ failure and mortality in patients with cardiogenic shock, needs to be investigated.

## Conclusion

This study demonstrates that after improvement of global hemodynamic parameters, microcirculatory hypoperfusion might still be present in the majority of patients admitted with cardiogenic shock. To some extent, microcirculation was improved by enoximone, but not by dobutamine or norepinephrine. These findings add to the concept that therapy may potentially be better tailored by assessment and optimization of microcirculation in the setting of acute cardiac care. Interventions directed at improving microvascular perfusion, such as inodilators (enoximone), and/or nitrates [20], [25] or ultimately mechanical circulatory support [37], may eventually help bridge the gap between improved hemodynamics and the dismal patient outcome in cardiogenic shock. Whether monitoring of tissue microcirculation optimizes current treatment strategies in patients with severe heart failure and whether such a strategy will favourably affect outcome, warrants further investigation.
